# Factors Associated With 30-Day Functional Outcome and Mortality Following Spontaneous Intracerebral Hemorrhage in Bangladesh: A Prospective Two-Center Cohort Study

**DOI:** 10.7759/cureus.115816

**Published:** 2026-09-05

**Authors:** S. M. Arafath Amin, Pijush Paul, Mizanur Rahman, Ashrafuzzaman Khan, Md. Raknuzzaman, Peyel A Khan, Mohiuddin Ahmed, Tasneem Jerin, Kazi G Ahmad, Md. Shamsuzzaman

**Affiliations:** 1 Neurology, National Institute of Neurosciences and Hospital, Dhaka, BGD; 2 Interventional Neurology, National Institute of Neurosciences and Hospital, Dhaka, BGD; 3 Neurology, Dhaka Medical College and Hospital, Dhaka, BGD; 4 Interventional Neuroradiology, National Institute of Neurosciences and Hospital, Dhaka, BGD; 5 Neurology, Dhaka Medical College, Dhaka, BGD; 6 Palliative Medicine, Palliative Care Society of Bangladesh, Dhaka, BGD

**Keywords:** aspiration pneumonia, functional outcome, hypernatremia, intracerebral hemorrhage, mortality, prognostic factors

## Abstract

Background

Spontaneous intracerebral hemorrhage remains a major cause of early mortality and disability worldwide, with a disproportionate burden in low- and middle-income countries such as Bangladesh. However, prospective population-specific data identifying short-term prognostic factors are limited. This study aimed to examine the clinical, radiological, and in-hospital factors associated with 30-day functional outcome and early mortality in a selected two-center cohort of Bangladeshi patients with spontaneous intracerebral hemorrhage.

Methods

This prospective cohort study was conducted in two tertiary referral centers in Dhaka between February 2021 and January 2023. Adult patients with first-ever spontaneous intracerebral hemorrhage confirmed by computed tomography and presenting within 72 hours were enrolled. Eligibility was restricted to patients with a baseline NIHSS of 5-15, defining a selected, moderate-severity cohort. Baseline demographic, clinical, laboratory, and radiological variables were recorded. Functional outcome at 30 days was assessed using the modified Rankin Scale. Unfavorable functional outcome (mRS 4-6, which includes death) was the primary outcome, and 30-day mortality was a secondary outcome. Multivariable logistic regression was used to examine factors associated with unfavorable outcomes, and Cox proportional hazards models were applied to examine factors associated with 30-day mortality.

Results

Among 220 patients, 72 (32.7%) died within 30 days, and 130 (59.1%) had unfavorable outcomes at 30 days. Overall, 90 (40.9%) patients had a favorable outcome (mRS 0-3) and 130 (59.1%) had an unfavorable outcome (mRS 4-6), the latter including the 72 deaths. Increasing age was independently associated with poor functional outcome (adjusted OR 1.07 per year; 95% CI 1.04-1.11; p < 0.001). Hemiparesis at admission (OR 7.05; 95% CI 1.65-30.11; p = 0.008), aspiration pneumonia (OR 4.63; 95% CI 1.64-13.10; p = 0.004), and hypernatremia (OR 14.34; 95% CI 2.26-91.16; p = 0.005) were also associated with unfavourable outcome, although aspiration pneumonia and hypernatremia are post-admission complications rather than baseline predictors. For mortality, older age (adjusted HR 1.02; 95% CI 1.00-1.04; p = 0.029) and higher baseline NIHSS (HR 1.23; 95% CI 1.09-1.39; p = 0.001) were independently associated with death, whereas the in-hospital complications aspiration pneumonia (HR 3.34; 95% CI 1.94-5.74; p < 0.001) and hypernatremia (HR 3.30; 95% CI 1.99-5.82; p < 0.001) were associated with higher mortality but, as time-dependent events, should not be interpreted as baseline predictors.

Conclusion

Advancing age, greater neurological severity, and in-hospital complications such as aspiration pneumonia and hypernatremia were associated with short-term functional outcome and mortality. These associations may help identify high-risk patients, but because this was an observational study of a selected cohort in which in-hospital complications were analyzed as fixed covariates, the findings should not be interpreted as evidence that management of these complications improves outcomes.

## Introduction

Spontaneous intracerebral hemorrhage (ICH) represents a formidable global health challenge, constituting a severe subtype of stroke with persistently high mortality and profound long-term disability rates [1,2]. Worldwide, ICH is responsible for a significant proportion of stroke-related deaths and disability-adjusted life years (DALYs) [3]. The burden is particularly disproportionate in low- and middle-income countries (LMICs), where ICH incidence is often higher, and outcomes are markedly worse compared to high-income settings [3,4]. In Bangladesh, specifically, ICH is a leading cause of mortality and disability, yet comprehensive epidemiological data and locally validated prognostic models remain limited [1,5]. This critical evidence gap hinders optimal patient management and resource allocation in low-resource neurology settings [1].

Early and accurate prognostication of short-term functional outcomes following ICH is clinically imperative [1,6]. Such predictions are crucial for informing immediate clinical decisions, including intensive care unit (ICU) triage and allocation of scarce resources, especially in settings with constrained critical care capacity [1,7]. Moreover, precise prognostication facilitates meaningful family counseling regarding goals of care and anticipated recovery trajectories [1,7]. It also enables the timely implementation of strategies to prevent or mitigate secondary complications, thereby optimizing patient care before irreversible neurological deterioration occurs [2,8].

Prognostic frameworks for ICH integrate several categories of determinants. Clinical severity markers are consistently associated with adverse outcomes [2,3,8]. These include advanced age [9], lower Glasgow Coma Scale (GCS) scores on admission [10], and a higher National Institutes of Health Stroke Scale (NIHSS) score [11].

Radiological features such as the presence of intraventricular hemorrhage are consistently associated with poor functional outcomes and mortality [12]. Hydrocephalus, often a consequence of ventricular involvement, independently contributes to increased morbidity and mortality [13]. In addition, in-hospital complications, including aspiration pneumonia, electrolyte imbalance, and acute kidney injury, may significantly influence short-term outcomes following intracerebral hemorrhage [14].

To the best of our knowledge, prospective data on predictors of intracerebral hemorrhage (ICH) outcomes specifically in Bangladeshi populations are limited and, although several local studies exist, they have generally not separated baseline admission factors from post-admission in-hospital complications or reported internally validated prognostic models, despite numerous international studies identifying clinical, radiological, and in-hospital factors influencing prognosis. Recent prospective cohort studies in Bangladesh have found that higher age, lower GCS scores on admission, larger hematoma volume, presence of intraventricular hemorrhage (IVH), midline shift, hydrocephalus, and medical (non-surgical) management independently predict poorer functional outcomes and higher mortality at 30 to 90 days post-ICH [1,8,15]. The widely used ICH score has been validated locally as an effective tool for early risk stratification, with higher scores correlating strongly with increased 30-day mortality and poor functional outcomes [1,8]. Admission hyponatremia and elevated blood glucose levels have also been associated with worse short-term disability and mortality in Bangladeshi patients [16]. Additionally, nosocomial pneumonia and blood glucose levels at admission further modulate outcomes, highlighting the importance of managing in-hospital complications [15]. These findings underscore the need for population-specific prognostic models incorporating clinical status, imaging features, biochemical markers, and treatment modalities to optimize care delivery in resource-limited settings like Bangladesh [8,15]. This study, therefore, aims to address this critical knowledge gap by prospectively investigating the clinical, radiological, and in-hospital factors associated with 30-day functional outcome and early mortality in a selected two-center cohort of patients with spontaneous intracerebral hemorrhage in Bangladesh, while distinguishing baseline admission factors from post-admission in-hospital complications.

## Materials and methods

Study design and setting

This prospective cohort study was carried out over a two-year period from February 2021 to January 2023 in two tertiary referral centers in Dhaka, Bangladesh: the Department of Neurology at Dhaka Medical College and Hospital and the National Institute of Neurosciences and Hospital. Both institutions serve as major referral centers for acute stroke care and manage a high volume of intracerebral hemorrhage cases.

Study population

Adult patients (≥18 years) admitted with a diagnosis of spontaneous intracerebral hemorrhage were screened for eligibility. Intracerebral hemorrhage was confirmed by non-contrast computed tomography of the brain. Only patients presenting within 72 hours of symptom onset were considered for inclusion to ensure accurate assessment of early clinical and laboratory parameters.

Inclusion criteria

Patients were eligible if they were aged 18 years or older, had a first-ever spontaneous intracerebral hemorrhage, and had a baseline National Institutes of Health Stroke Scale score between 5 and 15 at admission. Both male and female patients were included.

Exclusion criteria

Patients were excluded if the hemorrhage was secondary to trauma, ischemic stroke with hemorrhagic transformation, or subarachnoid hemorrhage. Individuals who had undergone prior neurosurgical intervention for the index event were not included. Patients receiving antiplatelet or anticoagulant therapy, those with known bleeding disorders, recent acute or chronic infection within four weeks prior to the event, or severe systemic comorbidities such as advanced chronic kidney disease, congestive heart failure, chronic liver disease, respiratory failure, metabolic encephalopathy, malignancy, or pregnancy were also excluded (Table 1).

**Table 1:** Eligibility criteria for study participation

Eligibility category	Criterion
Inclusion	Age ≥18 years
Inclusion	First-ever spontaneous intracerebral hemorrhage
Inclusion	Diagnosis confirmed by non-contrast computed tomography of the brain
Inclusion	Presentation within 72 hours of symptom onset
Inclusion	Baseline National Institutes of Health Stroke Scale score of 5–15
Inclusion	Male or female sex
Exclusion	Intracerebral hemorrhage secondary to trauma
Exclusion	Hemorrhagic transformation of an ischemic stroke
Exclusion	Subarachnoid hemorrhage
Exclusion	Previous neurosurgical intervention for the index hemorrhagic event
Exclusion	Current antiplatelet therapy
Exclusion	Current anticoagulant therapy
Exclusion	Known bleeding disorder
Exclusion	Acute or chronic infection within the preceding four weeks
Exclusion	Advanced chronic kidney disease
Exclusion	Congestive heart failure
Exclusion	Chronic liver disease
Exclusion	Respiratory failure
Exclusion	Metabolic encephalopathy
Exclusion	Malignancy
Exclusion	Pregnancy

Sample size and enrollment

Participants were recruited using a consecutive sampling approach. All patients who presented during the study period were screened against the predetermined eligibility criteria, and those who fulfilled the requirements were enrolled after written informed consent had been obtained. The minimum sample size was determined using the single-population proportion formula:

$n = \frac{Z^{2}pq}{d^{2}}$

For the calculation, a 95% confidence level was applied, corresponding to a Z value of 1.96, with an allowable margin of error of 5%. The anticipated proportion of an unfavorable functional outcome at 30 days was considered to be 80.8% (p = 0.808), while the complementary proportion was 19.2% (q = 0.192). These estimates were derived from a previous Bangladeshi cohort reported by Tapu et al., in which 19.2% of patients with spontaneous intracerebral hemorrhage achieved a favorable functional outcome at one month [17]. Based on these assumptions, the minimum required sample was approximately 239 participants and was rounded to 240. During the study period, 250 eligible patients were consecutively enrolled, thereby exceeding the calculated requirement. Of these, 30 participants (12.0%) were lost to follow-up, leaving 220 patients for the final analysis.

Data collection procedures

Baseline demographic and clinical information was recorded using a structured data collection form. Variables included age, sex, residence, and vascular risk factors such as hypertension, diabetes mellitus, smoking status, and dyslipidemia. Neurological assessment at admission included scoring on the Glasgow Coma Scale and the National Institutes of Health Stroke Scale. Blood pressure measurements were recorded on arrival. Focal motor deficit (hemiparesis) was recorded at admission as the presence of reduced limb power contralateral to the hemorrhage. The GCS and NIHSS were assessed at the time of admission; because these scores can fluctuate during the early hours after ICH onset, the admission values may not capture subsequent neurological change. All patients underwent standard laboratory investigations, including blood glucose, serum electrolytes, renal function tests, and other routine biochemical parameters as per institutional protocol.

The Glasgow Coma Scale was used to assess the level of consciousness, based on eye, verbal, and motor responses, with lower scores indicating greater neurological impairment. Although this combined scoring system offers a comprehensive evaluation of brain function, its application may require additional time, which can delay urgent clinical interventions. Initial stroke severity was measured using the National Institutes of Health Stroke Scale, a standardized tool scored from 0 to 42, where higher scores indicate more severe neurological deficits [18].

Radiological assessment

Non-contrast CT brain imaging was performed at admission to confirm the diagnosis and to evaluate hematoma characteristics. Hemorrhage location was categorized as supratentorial or infratentorial, and further classified by laterality. The presence of intraventricular hemorrhage, hydrocephalus, midline shift, and other structural features was documented by radiology specialists blinded to outcome status. Hematoma volume was not quantified, as standardized volumetric software was not available at the participating centers during the study period; this is acknowledged as a limitation, since hematoma volume is an important prognostic determinant and a potential confounder of the observed associations.

In-hospital monitoring and management

All patients received standard medical management according to contemporary stroke care guidelines. Blood pressure was controlled with oral or intravenous antihypertensive agents as indicated. Airway protection, nutritional support, and prevention of complications such as deep vein thrombosis and aspiration were ensured. Patients with respiratory compromise or severe neurological deterioration were transferred to intensive care when required. In-hospital complications were actively monitored and recorded, including aspiration pneumonia, electrolyte imbalance such as hypernatremia, acute kidney injury, and delirium, using predefined clinical and laboratory criteria detailed below.

In-hospital complications were diagnosed using predefined criteria. Aspiration pneumonia was diagnosed on the basis of new respiratory signs and symptoms together with compatible chest radiographic findings following a witnessed or suspected aspiration event. Hypernatremia was defined as a serum sodium concentration above 145 mmol/L recorded at any point during the hospital stay; admission and hospital-acquired episodes were not separately distinguished. Acute kidney injury was defined according to the Kidney Disease: Improving Global Outcomes (KDIGO) serum-creatinine criteria. Delirium was identified through daily clinical assessment for an acute, fluctuating disturbance of attention and awareness. All events were adjudicated by the treating clinical team using these criteria.

Outcome assessment

Functional outcome was assessed using the modified Rankin Scale. This scale classifies functional ability into seven levels, ranging from 0, indicating no residual symptoms, to 6, indicating death, with intermediate grades reflecting increasing degrees of disability. For analytical purposes, scores of 0 to 3 were defined as favorable outcomes, whereas scores of 4 to 6 were considered unfavorable [19]. Mortality within 30 days was also documented. The 30-day modified Rankin Scale was assessed by trained physicians through a structured in-person or telephone interview at day 30 (± 3 days); where a patient was unable to respond, information was obtained from a primary caregiver. Although the 0-2 versus 3-6 dichotomy is more commonly used, the 0-3 versus 4-6 cut point was prespecified for this cohort to correspond to the threshold for functional dependence requiring assistance; the full ordinal mRS distribution and ordinal (shift) analysis were not evaluated.

Statistical analysis

Data were analyzed using SPSS Statistics for Windows, version 26.0 (IBM Inc., Armonk, NY, USA). Continuous variables were tested for normality and expressed as mean with standard deviation when normally distributed, or as median with interquartile range when distribution was skewed. Categorical variables were presented as frequencies and percentages. Comparisons between favorable and unfavorable outcome groups were performed using an independent samples t-test for normally distributed continuous variables and the Mann-Whitney U test for non-normally distributed variables. Chi-squared test or Fisher's exact test was applied for categorical variables as appropriate. Variables showing statistical significance in univariate analysis were entered into multivariable binary logistic regression models to examine factors independently associated with unfavorable 30-day functional outcome. Adjusted odds ratios with 95 percent confidence intervals were calculated. Because only variables reaching significance in univariate analysis were carried forward, this data-driven selection may not correspond to a fully prespecified adjustment set; the models were not internally validated, and residual confounding from unmeasured baseline variables (including hematoma volume) cannot be excluded. To evaluate factors associated with 30-day mortality, Cox proportional hazards regression analysis was performed. The time origin was the date of hospital admission, survival time was measured as days from admission to death, and patients who survived to day 30 or were lost to follow-up were censored at their last recorded assessment. Hazard ratios with 95 percent confidence intervals were reported. In-hospital complications, which arise after admission, were entered as time-fixed covariates rather than time-varying covariates; this is addressed as a limitation. All statistical tests were two-tailed, and a p-value less than 0.05 was considered statistically significant.

Ethical approval

The study protocol was reviewed and approved by the Ethical Review Committee of Dhaka Medical College (approval no: ERC-DMC/ECC/2021/10). The study was conducted in accordance with the principles of the Declaration of Helsinki. Written informed consent was obtained from each patient. For patients who lacked decision-making capacity owing to impaired consciousness, written informed consent was obtained from a legally authorized representative or next of kin.

## Results

Patients aged 60 years or younger were significantly more common in the favorable outcome group than in the unfavorable outcome group (77 (85.6%) vs. 62 (47.7%); χ² (1) = 32.78, p-value < 0.001). The association between age group and 30-day functional outcome showed a moderate effect size (Cramér's V = 0.386). Smoking was also significantly associated with functional outcome, with a higher proportion of smokers observed among patients with unfavorable outcomes than among those with favorable outcomes (70 (53.8%) vs. 36 (40.0%); χ² (1) = 4.08, p-value = 0.043), although the effect size was small (Cramér's V = 0.136). No statistically significant differences were observed between the outcome groups regarding sex, residence, hypertension, diabetes, or dyslipidemia (Table 2).

**Table 2:** Sociodemographic characteristics and vascular risk factors according to 30-day functional outcome (N=220) Data are presented as mean ± standard deviation or frequency (percentage), as appropriate. The independent-samples t-test was used for continuous variables, while the chi-squared test or Fisher's exact test was used for categorical variables, as appropriate. A p-value of <0.05 was considered statistically significant.

Variable	Favorable (n=90)	Unfavorable (n=130)	χ² (df)	Cramér's V	p-value
Age ≤60 yrs	77 (85.6%)	62 (47.7%)	32.78 (1)	0.386	<0.001
Male sex	45 (50.0%)	69 (53.1%)	0.20 (1)	0.030	0.653
Rural residence	58 (64.4%)	87 (66.9%)	0.15 (1)	0.026	0.703
Hypertension	74 (82.2%)	113 (86.7%)	0.92 (1)	0.065	0.337
Smoking	36 (40.0%)	70 (53.8%)	4.08 (1)	0.136	0.043
Diabetes	15 (16.7%)	15 (11.5%)	1.19 (1)	0.073	0.276
Dyslipidaemia	11 (12.2%)	14 (10.8%)	0.11 (1)	0.023	0.738

At admission, patients with unfavorable 30-day functional outcomes had significantly lower Glasgow Coma Scale scores than those with favorable outcomes (9.8 ± 0.9 vs. 10.1 ± 0.9; t (218) = 2.06, p-value = 0.041; Cohen's d = 0.333). They also had significantly higher National Institutes of Health Stroke Scale scores (7.6 ± 2.0 vs. 6.8 ± 1.8; t (218) = 2.91, p-value = 0.004; Cohen's d = −0.416) and higher systolic blood pressure (168.1 ± 24.1 vs. 159.2 ± 30.8 mmHg; t (218) = 2.38, p-value = 0.018; Cohen's d = −0.329). These differences represented small to moderate effect sizes. The absolute between-group differences in GCS (0.3 points) and blood pressure were modest, and their clinical relevance should be interpreted with caution alongside their statistical significance. Diastolic blood pressure did not differ significantly between the groups (t (218) = 1.69, p-value = 0.092). Radiological findings were also associated with functional outcome. Supratentorial hematoma was more frequent in the unfavorable group than in the favorable group (119 (91.5%) vs. 73 (81.1%); χ² (1) = 5.21, p-value = 0.023). Similarly, left-sided lesions (74 (56.9%) vs. 25 (27.8%); χ² (1) = 18.25, p-value < 0.001), intraventricular hemorrhage (83 (63.8%) vs. 40 (44.4%); χ² (1) = 8.12, p-value = 0.004) and hydrocephalus (58 (44.6%) vs. 25 (27.8%); χ² (1) = 6.42, p-value = 0.011) were significantly more common among patients with unfavorable outcomes. Among these radiological characteristics, left-sided lesions demonstrated the strongest association with outcome, with a small-to-moderate effect size (Cramér's V = 0.288) (Table 3).

**Table 3:** Admission clinical and radiological characteristics according to 30-day functional outcome (N=220) GCS - Glasgow Coma Scale; NIHSS - National Institutes of Health Stroke Scale; SBP - systolic blood pressure; DBP - diastolic blood pressure; IVH - intraventricular hemorrhage; SD - standard deviation

Characteristic	Favorable (n=90)	Unfavorable (n=130)	Test statistic (df)	Effect size	p-value
GCS, mean ± SD	10.1 ± 0.9	9.8 ± 0.9	t = 2.06 (218)	Cohen’s d = 0.333	0.041
NIHSS, mean ± SD	6.8 ± 1.8	7.6 ± 2.0	t = 2.91 (218)	Cohen’s d = −0.416	0.004
SBP, mmHg, mean ± SD	159.2 ± 30.8	168.1 ± 24.1	t = 2.38 (218)	Cohen’s d = −0.329	0.018
DBP, mmHg, mean ± SD	96.1 ± 14.9	92.6 ± 15.2	t = 1.69 (218)	Cohen’s d = 0.232	0.092
Supratentorial hematoma, n (%)	73 (81.1%)	119 (91.5%)	χ² = 5.21 (1)	Cramér’s V = 0.154	0.023
Left-sided lesion, n (%)	25 (27.8%)	74 (56.9%)	χ² = 18.25 (1)	Cramér’s V = 0.288	<0.001
Intraventricular hemorrhage, n (%)	40 (44.4%)	83 (63.8%)	χ² = 8.12 (1)	Cramér’s V = 0.192	0.004
Hydrocephalus, n (%)	25 (27.8%)	58 (44.6%)	χ² = 6.42 (1)	Cramér’s V = 0.171	0.011

Aspiration pneumonia occurred substantially more frequently among patients with unfavorable outcomes than among those with favorable outcomes (46 (35.4%) vs. 8 (8.9%); χ² (1) = 20.16, p-value < 0.001). This association demonstrated a moderate effect size (Cramér's V = 0.303). Hypernatremia was also significantly more common in the unfavorable outcome group (19 (14.6%) vs. 2 (2.2%); χ² (1) = 9.46, p-value = 0.002), with a small-to-moderate effect size (Cramér's V = 0.207). Acute kidney injury (36 (27.7%) vs. 16 (17.8%); p-value = 0.089) and delirium (23 (17.7%) vs. 8 (8.9%); p-value = 0.065) were numerically more frequent among patients with unfavorable outcomes, but the differences were not statistically significant (Table 4).

**Table 4:** In-hospital complications according to 30-day functional outcome (N=220) AKI - acute kidney injury

Complication	Favorable (n=90)	Unfavorable (n=130)	χ² (df)	Cramér’s V	p-value
Aspiration pneumonia	8 (8.9%)	46 (35.4%)	20.16 (1)	0.303	<0.001
Hypernatremia	2 (2.2%)	19 (14.6%)	9.46 (1)	0.207	0.002
AKI	16 (17.8%)	36 (27.7%)	2.90 (1)	0.115	0.089
Delirium	8 (8.9%)	23 (17.7%)	3.40 (1)	0.124	0.065

After adjustment for potential confounders in Table 5, increasing age remained independently associated with unfavorable functional status at 30 days (adjusted OR 1.07 per year; 95% CI 1.04-1.11; p < 0.001). Hemiparesis at admission (OR 7.05; 95% CI 1.65-30.11; p = 0.008), aspiration pneumonia (OR 4.63; 95% CI 1.64-13.10; p = 0.004) and hypernatremia (OR 14.34; 95% CI 2.26-91.16; p = 0.005) were also independently associated with unfavorable outcomes.

**Table 5:** Multivariable binary logistic regression analysis of factors associated with unfavourable 30-day functional outcome (N=220) OR - odds ratio; CI - confidence interval

Variables	Adjusted OR	95% CI	p-value
Age	1.07	1.04–1.11	<0.001
Hemiparesis	7.05	1.65–30.11	0.008
Aspiration pneumonia	4.63	1.64–13.10	0.004
Hypernatremia	14.34	2.26–91.16	0.005

In survival analysis, age was associated with higher 30-day mortality (adjusted HR 1.02; 95% CI 1.00-1.04; p = 0.029). Baseline NIHSS score was independently associated with death (HR 1.23; 95% CI 1.09-1.39; p = 0.001), while aspiration pneumonia (HR 3.34; 95% CI 1.94-5.74; p < 0.001) and hypernatremia (HR 3.30; 95% CI 1.99-5.82; p < 0.001) were associated with more than a threefold higher mortality hazard; because these complications develop after admission and were modeled as time-fixed covariates, their hazard ratios should be interpreted as associations rather than baseline or causal effects (Table 6).

**Table 6:** Multivariable Cox proportional hazards regression analysis of factors associated with 30-day mortality (N=220) HR - hazard ratio; CI - confidence interval; NIHSS - National Institutes of Health Stroke Scale

Variables	Adjusted HR	95% CI	p-value
Age	1.02	1.00–1.04	0.029
NIHSS	1.23	1.09–1.39	0.001
Aspiration pneumonia	3.34	1.94–5.74	<0.001
Hypernatremia	3.30	1.99–5.82	<0.001

## Discussion

This study examined the clinical, radiological, and in-hospital factors associated with 30-day functional outcome and early mortality among patients with spontaneous intracerebral hemorrhage in Bangladesh. We observed that 130 (59.1%) patients experienced unfavorable outcomes, and the 30-day mortality rate was 72 (32.7%). Additionally, advancing age, higher baseline neurological severity, aspiration pneumonia, and hypernatremia emerged as the factors most strongly associated with these outcomes.

In our cohort, older age and markers of neurological injury severity were strongly associated with poor 30-day outcomes. This finding is consistent with international data. For example, an analysis of the multinational INTERACT2 trial found patients over 75 years had over four times the odds of death or major dependency at 90 days compared to those under 52, even after adjusting for confounders [9]. Advanced age likely reflects diminished physiological reserve and a higher prevalence of comorbidities, limiting recovery potential. Neurologic status at presentation has long been recognized as prognostic: each incremental drop in GCS or rise in stroke severity correlates with exponentially higher odds of unfavorable outcome [20]. A recent prospective study from China demonstrated that for each one-point increase in NIHSS, the odds of three-month disability rose by ~21%, and increasing age independently worsened outcomes in parallel [20]. In our cohort, each one-point increase in NIHSS was associated with a 23% higher hazard of 30-day mortality (adjusted HR 1.23), comparable to the ~21% increase in disability risk reported in that Chinese cohort.

Key radiological features of the hemorrhage significantly influenced outcomes in our study. Notably, IVH and secondary hydrocephalus were associated with poorer outcomes in univariate analysis, although these associations did not remain statistically significant after multivariable adjustment. This aligns with prior reports that extension of blood into the ventricles portends a worse prognosis. In a recent multivariable analysis, the presence of IVH increased the odds of three-month disability by nearly fourfold [20]. Likewise, a 2023 meta-analysis confirmed that ICH patients who develop hydrocephalus have dramatically higher mortality risk - approximately two to four times greater than those without - and markedly lower odds of functional recovery [13]. The mass effect and elevated intracranial pressure associated with ventricular obstruction provide a mechanistic explanation for these outcomes. Lesion location and laterality also played a role. Our finding that left-hemisphere hematomas were associated with worse functional outcomes in univariate analysis may reflect the impact of dominant hemisphere damage on language and cognitive function, which can impede rehabilitation. A study has noted contradictory results (e.g., right-sided ICH linked to mood disturbances or neglect), but after adjusting for confounders like IVH volume, found no inherent survival difference by hemisphere [21].

Aspiration pneumonia was a frequent medical complication in our cohort and was strongly associated with poor 30-day outcomes. This finding resonates with stroke studies worldwide that identify stroke-associated pneumonia as a major contributor to early mortality and disability [22]. In patients with ICH, greater neurological severity, impaired consciousness, and swallowing dysfunction may increase the risk of aspiration pneumonia [15,22]. These findings support prompt dysphagia screening, appropriate positioning, early mobilization, and timely management of suspected aspiration pneumonia.

Hypernatremia during acute ICH care was associated with unfavorable 30-day outcomes. Patients who developed elevated serum sodium levels were more likely to have poor functional status, reflecting the association between disordered sodium homeostasis and critical illness severity. This result is in line with emerging evidence from critical care cohorts. Recent analyses of intensive care unit patients with ICH have shown that hypernatremia (often defined as Na >145 mmol/L) is a robust marker of mortality risk [23]. In another study, ICH patients with serum sodium >145 at discharge had nearly double the odds of dying in the next 30 days compared to normonatremic patients [24]. Our finding that hypernatremia portends poor outcome aligns with the concept that patients with the most severe brain injury (who often require aggressive osmotic therapy or develop neuroendocrine dysfunction) are the ones at highest risk of death and disability [23,24].

The history of cigarette smoking was associated with short-term unfavorable outcomes in this cohort. A comprehensive review concluded that beyond increasing ICH incidence, chronic smoking contributes to larger hematoma volumes, greater odds of hematoma expansion, and worsened mortality and functional outcomes after ICH [25]. Notably, a retrospective study in the United States found that while unadjusted analyses suggested recent smokers had higher rates of 90-day independence than nonsmokers, those differences vanished after accounting for baseline factors [26]. This implies that any transient neuroprotective effects of nicotine are outweighed by the long-term vascular damage and comorbid risk that smokers carry.

Interestingly, acute kidney injury (AKI) and delirium were not significantly associated with short-term functional status in unadjusted analyses in our study. This is somewhat unexpected given prior studies linking each to a worse prognosis in stroke populations. AKI has been associated with increased mortality and neurological deterioration following spontaneous ICH [27,28]. Literature suggests that ICH patients who develop AKI have higher in-hospital mortality and greater risk of neurological decline compared to those maintaining renal stability [27,28]. Likewise, post-stroke delirium is widely associated with poor outcomes. A meta-analysis of stroke patients found that delirium was correlated with increased 30-day and 90-day mortality and a higher likelihood of dependence at discharge [29]. Because AKI and delirium were not entered into an adjusted model, no conclusion can be drawn about their independent prognostic value; the unadjusted patterns observed here require confirmation in adequately powered studies using appropriate multivariable adjustment.

Results from our study further demonstrated that advanced age and higher NIHSS were independently associated with increased 30-day mortality. Several prior studies have reported similar findings, identifying age and baseline neurological severity as strongly associated with early death following intracerebral hemorrhage [30]. In addition, aspiration pneumonia and hypernatremia were associated with increased mortality in our cohort. These observations are consistent with previous literature showing that stroke-associated pneumonia markedly worsens short-term survival [15,22], while sodium imbalance, particularly hypernatremia, is associated with increased mortality following primary ICH [23,24]. Collectively, these findings reinforce the combined impact of primary neurological injury and secondary medical complications on early mortality in ICH patients [15,22,24].

This study had several strengths, including its prospective design, consecutive recruitment, CT-confirmed diagnosis, enrolment from two high-volume tertiary centers, use of standardized neurological and functional scales, and assessment of both disability and mortality in an under-studied low- and middle-income population. Several limitations should be emphasized. First, aspiration pneumonia and hypernatremia are in-hospital complications that develop after the prognostic time origin; they were analyzed as fixed rather than time-dependent covariates, so their associations are susceptible to reverse causation and time-dependent bias and should be read as associations rather than independent or causal predictors, and the proportional-hazards assumption was not formally tested. Second, the cohort was highly selected-restricted to first-ever ICH presenting within 72 hours with a baseline NIHSS of 5-15 and excluding antiplatelet or anticoagulant users and several comorbidities-yielding a moderate-severity, hospital-based sample from two Dhaka centers that is not generalizable to the full ICH spectrum. Third, hematoma volume and other prognostic variables (pre-stroke mRS, exact site, admission glucose and sodium, and acute treatment exposures) were unavailable, so residual confounding cannot be excluded. Fourth, candidate variables were selected by univariate significance rather than a prespecified set; the models were not internally validated, and the wide confidence interval for hypernatremia indicates sparse-data instability; the results represent associations, not a validated prognostic model. The 220 analyzed patients fell below the calculated target (239-240), events per variable were limited, non-significant findings may reflect type II error, and several significant differences had small effect sizes. Fifth, 30 patients (12%) were lost to follow-up and handled by complete-case analysis without sensitivity analysis; the mRS was dichotomized rather than analyzed ordinally, and outcome assessors were not blinded. These findings are therefore best interpreted as hypothesis-generating and require confirmation in prospective, externally validated, multicentre studies.

## Conclusions

In this study, increasing age, higher baseline neurological severity, aspiration pneumonia, and hypernatremia were independently associated with unfavorable 30-day functional outcomes following spontaneous intracerebral hemorrhage. These factors were also associated with early mortality. These findings identify high-risk patients who may warrant closer monitoring; however, because this was an observational study of a selected hospital cohort in which post-admission complications were analyzed as fixed covariates, they should be interpreted as associations rather than evidence that targeted management improves outcomes. Further prospective, externally validated, multicenter studies in broader, unselected populations are warranted to confirm these associations and to distinguish baseline prognostic factors from time-dependent in-hospital complications.
